# Platelet Deposition Onto Vascular Wall Regulated by Electrical Signal

**DOI:** 10.3389/fphys.2021.792899

**Published:** 2021-12-23

**Authors:** Mingyan Wang, Wei Zhang, Zhi Qi

**Affiliations:** ^1^Department of Basic Medical Sciences, School of Medicine, Xiamen University, Xiang’an Nan Lu, Xiamen, China; ^2^Xiamen Institute of Cardiovascular Diseases, The First Affiliated Hospital of Xiamen University, School of Medicine, Xiamen University, Xiamen, China

**Keywords:** platelet, deposition, primary hemostasis, transvascular electric potential, vascular injury

## Abstract

Platelets deposition at the site of vascular injury is a key event for the arrest of bleeding and for subsequent vascular repair. Therefore, the regulation of platelet deposition onto the injured site during the process of platelet plug formation is an important event. Herein, we showed that electrical signal could regulate the deposition of platelets onto the injured site. On the one hand, the area of platelet deposition was reduced when the cathode of the applied electric field was placed at the injured site beforehand, while it was increased when the anode was at the site. On the other hand, if a cathode was placed at the injured site after the injury, the electrical signal could remove the outer layer of the deposited platelets. Furthermore, an electric field could drive rapid platelet deposition onto the blood vessel wall at the site beneath the anode even in uninjured blood vessels. Platelet deposition could thus be manipulated by externally applied electric field, which might provide a mechanism to drive platelet deposition onto the wall of blood vessels.

## Introduction

Living cell responding to imposed electric field (EF) by migrating toward cathode or anode is known as electrotaxis or galvanotaxis. After its discovery one century ago (Sato et al., 2007), various cell types have been reported to migrate under EF. These cells include cancer cells (Djamgoz et al., 2001; Yan et al., 2009; Sun et al., 2012; Pu et al., 2015), human primary T cells (Arnold et al., 2019), lymphocytes (Lin et al., 2008), macrophages (Hoare et al., 2016; Sun et al., 2019), neural stem cells (Feng et al., 2012), and oligodendrocyte precursor cells (Li et al., 2015). Moreover, it has been shown that EF plays crucial roles in many physiological and pathophysiological processes, such as wound healing (Zhao et al., 2006), cell division (Zhao et al., 1999), stem cell migration (Feng et al., 2012; Funk, 2015), nerve regeneration (Song et al., 2004), metastatic process (Djamgoz et al., 2001), T cell functions (Arnold et al., 2019), and macrophage phagocytic uptake (Hoare et al., 2016; Sun et al., 2019). For example, under the stimulus of EF, cells could migrate in a specific direction to repair tissue damage (Zhao et al., 2006). Therefore, exogenous EF has been widely applied to accelerate the wound-healing process (Balakatounis and Angoules, 2008; Hunckler and de Mel, 2017; Luo et al., 2021).

Sialic acid (*N*-acetyl-neuraminic acid), a negatively charged sugar and constituent of many glycoproteins and gangliosides, is known to confer the bulk of negative charges to mammalian cell surfaces. Platelets contain sialic acid (Born and Palinski, 1985; Crook, 1991), which makes the platelet has an overall electronegative charge on its surface (Crook and Crawford, 1988). Due to this property, an electric field has been exerted on platelets suspended in the electrolytic medium for electrophoresis, which can be retrieved from the study by Abramson (1928). These electrophoretic investigations of platelets have been carried out to determine the ionic groups responsible for the charge (Seaman and Vassar, 1966; Nomura and Takagi, 1974; Menashi et al., 1981), to judge the effect of drugs on platelets (Betts et al., 1968; Florence and Rahman, 2011; Wang et al., 2017), or to estimate the electrophoretic mobility of platelets in patients with various clinical conditions (Grottum, 1968; Karppinen and Halonen, 1970). In addition, an electric field has been applied onto a cylindrical chamber or capillary to study interactions between small molecules and platelets during their aggregation (Seaman and Vassar, 1966; Wang et al., 2015, 2017). However, whether the electric field could direct platelets toward the vascular wall *in vivo* to facilitate platelet deposition over there, especially in an injured blood vessel, has not been studied.

The main physiological function of platelets is to participate in primary hemostasis through three distinct steps: adhesion, activation, and aggregation. During these steps, a chain of events is triggered, ultimately leading to the formation of a platelet plug to prevent blood loss as rapidly as possible (Ruggeri and Mendolicchio, 2007; Versteeg et al., 2013). The first step to form the platelet plug is that the circulating platelets need to deposit to the exposed injured surface. Given their negative surface charge, it could be reasoned that EF might act as a potential guidance cue to regulate the deposition of platelet onto the injured surface of blood vessels. To test this hypothesis, we conducted experiments to investigate whether an applied EF could regulate the deposition of platelets onto the blood vessels.

## Materials and Methods

### Animals

Male KM mice (weighing 20–30 g, Xiamen University Laboratory Animal Center) were anesthetized by intraperitoneal injection of 1.5% pentobarbital sodium. Animal care and experiments were performed in accordance with the procedures approved by the Animal Care and Use Committee of Xiamen University.

### Experimental Materials

Pentobarbital sodium was from Sinopharm Chemical Reagent (China). 5, 6-carboxyfluorescein-succinimidyl-ester (CFSE) was from Invitrogen (Molecular Probes, Eugene, OR, United States). Poly-dimethyl-siloxane (PDMS; Sylgard 184, Dow Silicones, Midland, MI, United States) was from Dow Silicones Co. (United States). Stainless steel microwires were from Shanghai Lei Yu Materials Co. Ltd. (China). Neuraminidase was from Shanghai Canspec S&T Co. LTD (China).

### Assessment of Platelet Deposition by Intravital Microscopy

Platelet deposition in the mesenteric vessels (diameter from 20 to 80 μm) of anesthetized mice was visualized using an inverted fluorescence microscope (IX7.1, Olympus, Japan) equipped with a digital camera. Images were collected as time series with acquisition rates of 10 frames/s. Images were analyzed using ImageJ (NIH, Bethesda, MD, United States). Vessel wall injury was performed mechanically by puncturing the blood vessel wall with a glass microelectrode. The starting time of punctured injury was from the moment when the microelectrode was rapidly withdrawn from the puncture site.

### Transvascular Electric Potential Measurement

**FIGURE 1:**
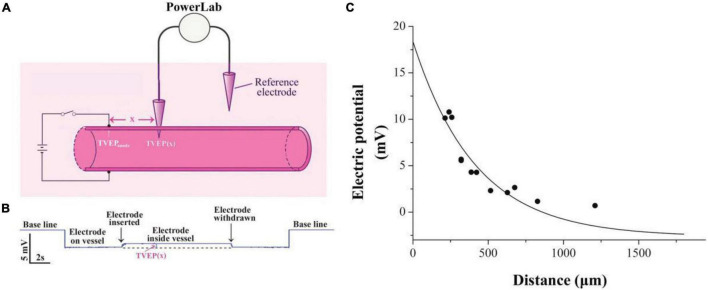
Exponential decay of applied electrical potential along with the blood vessel. **(A)** Schematic diagram showing measurement of TVEP(x) in mesenteric vessels after applying EF on the vessel wall. TVEP(x) was obtained by PowerLab system when the microelectrode penetrated the vessel. **(B)** Representative voltage trace for the measurement. Penetration of the wall caused a sharp upward deflection, indicating that TVEP(x) is positive relative to the reference electrode in the bath. **(C)** TVEP(x) decays along with the blood vessel wall. TVEP was plotted against distance from the tip of the potential applying microelectrode. The curve was drawn according to the least square procedure (Levenberg-Marquardt algorithm) to fit the data to the equation: TVEP(x) = TVEP_anode_*exp-x/λ + TVEPo.

Transvascular electric potential (TVEP) is defined as the potential inside the blood vessel relative to the reference electrode outside (Figure 1A). Its measurement is essentially the same as described previously (Fajac et al., 1998) with slight modifications. Glass microelectrodes were pulled from thin-walled borosilicate capillary tubes using a micropipette puller (model P-10, Narishige) with resistance in a range of 2–5 MΩ (tip diameter, 2–4 μm) when filled with Hanks’ balanced salt solution (HBSS; Gibco, in mM): 1.26 CaCl_2_, 0.49 MgCl_2_, 0.41 MgSO_4_, 5.33 KCl, 0.44 KH_2_PO_4_, 4.17 NaHCO_3_, 137.9 NaCl, 0.34 Na_2_HPO_4_, and 5.56 D-glucose. Silver wire, the end of which was covered by a layer of AgCl, was inserted into the tubes to make Ag/AgCl microelectrodes. The TVEP was measured by a potential-measuring circuit that was connected to Bio Amp device PowerLab System (PowerLab 8/30; ADInstruments, Australia) through a pair of Ag/AgCl microelectrodes (Figure 1A). The tip of one microelectrode was placed outside the adventitia of the blood vessel to serve as the reference electrode. The tip of the other microelectrode that gently penetrated the intima of the vessel with the use of a micromanipulator served as the recording electrode. All data were acquired and transferred to a computer for further analysis by using Chart or Scope software (ADInstruments).

### Platelet Isolation and Transfer

To isolate platelets, blood was withdrawn from the inferior vena cava of anesthetized donor mice, and platelet-rich plasma was prepared through sequential centrifugation. Platelets were then pelleted from platelet-rich plasma and gently resuspended in Tyrode’s buffer (10 mmol/L HEPES, 12 mmol/L NaHCO_3_, pH 7.4, 137 mmol/L NaCl, 2.7 mmol/L KCl, 5 mmol/L glucose). Platelets were allowed to rest in Tyrode’s buffer for 30 min at room temperature. Subsequently, platelets were labeled with CFSE (peak absorbance, 492 nm; peak emission, 518 nm) (5 mmol/L; Invitrogen) and centrifuged at 2000 *g* for 15 min to obtain the labeled platelets as described previously (Devi et al., 2010). Then, the platelets were slowly infused intravenously and visualized by using fluorescence microscope.

### Fabrication of the Circular Microfluidic Channel

The microelectrode penetrable circular microfluidic channel with a diameter of 50 μm was fabricated according to a previous report (Qi et al., 2020). Briefly, a mixture of PDMS and its curing agent (10:1) was poured into an enclosed space, in which a stainless steel microwire with a diameter of 50 μm was aligned. After 30 min of heating under 75°C, the PDMS with the 50 μm microwire was placed into an ethanol pool for 12 h to swell slightly (swelling coefficient: 1.04) wetting the interface (Jia et al., 2008). Then, the wire in the PDMS was drawn out manually by applying a gentle force leading to the formation of a 50 μm circular microfluidic channel in the PDMS.

### Blood Sample

Blood samples of the mice were collected from the inferior vena cava of anesthetized donor mice and anticoagulated using heparin. The collected whole blood was perfused through the microfluidic channel using a syringe pump (model Ph.D 2000, Harvard Apparatus, United Kingdom). Prior to the sample perfusion, degassed PBS buffer was used to prime the channels to remove air bubbles. For neuraminidase-treated groups, the blood samples were incubated with neuraminidase (1 U/ml) for 30 min under 37°C.

### Statistical Analysis

All data were shown as mean ± SEM. Data were analyzed statistically by one-way ANOVA followed by the Bonferroni test for multiple comparisons with Origin7.0 (Microcal Software, Inc., Northampton, MA, United States). An unpaired Student’s *t*-test was used for two-group comparisons. Values of *p* < 0.05 were considered to be statistically significant.

## Results

### Exponential Decay of External Applied Electrical Potential Along With the Blood Vessel

Practically, it is very difficult to measure the electric potential directly beneath the anode. Therefore, the TVEP at different positions relative to the anode (Figures 1A,B) was measured for the estimation of the TVEP directly beneath the anode. For instance, each datum point in Figure 1C was the experimental measured TVEP at a corresponding distance from the tip of the anode when the outside adventitia is defined to be at ground potential (0 mV, reference electrode). We found that these potentials decayed exponentially along with the blood vessel according to the following equation:


(1) $$\mathrm{TVEP}\,\left(x\right)={\text{TVEP}}_{\text{anode}}*\exp-x/\lambda +\text{TVEPo}$$

where x is the distance from the anode to the potential measuring microelectrode, TVEP(x) is the TVEP at position x, TVEP_anode_ is the TVEP directly beneath the anode, and TVEPo is the TVEP of the blood vessel under physiological condition without any external applied EF. The spatial constant, λ, characterizes the attenuation and quantifies the extent of the spread of the TVEP_anode_ along with the blood vessel. For mesenteric vessels, TVEPo = − 2.65 ± 0.05 mV (*n* = 163) was obtained. The TVEP(x) was measured by the potential measuring microelectrode, which impaled the vessel wall at various positions along the vessel. Fitting the experimental data to Eq. 1, we obtained the spatial constant λ = 415.5 ± 68.1 μm. Based on this parameter, TVEP_anode_ for each of the experiments could be calculated by using Eq. 1. The EF in a given direction at any point is defined as the negative gradient of the potential in that direction at that point. Therefore, based on Eq. 1, the strength of the EF directly below the tip of the anode can be calculated as:


(2) $$\begin{array}{c} \text{EF}=-\text{d}\,\left\{\text{TVEP}\,\left(\text{x}\right)\right\}/\text{dx}\\ =-\text{d}\,\left\{{\text{TVEP}}_{\text{anode}}*\exp-x/\lambda +\text{TVEPo}\right\}/\text{dx}\\ ={\text{TVEP}}_{\text{anode}}*{\text{e}}^{-x/\lambda }/\lambda \end{array}$$

For example, the TVEP_anode_ for the experiment in Figure 1 is +18.4 mV, and thus, the EF directly below the tip of the anode is 44.3 mV/mm. This strength of the EF is within the range of EF that regulates the cellular signaling pathways (McCaig et al., 2005, 2009).

### Electrical Signal Regulates the Deposition of Platelets Onto the Surface of Injured Blood Vessels

**FIGURE 2:**
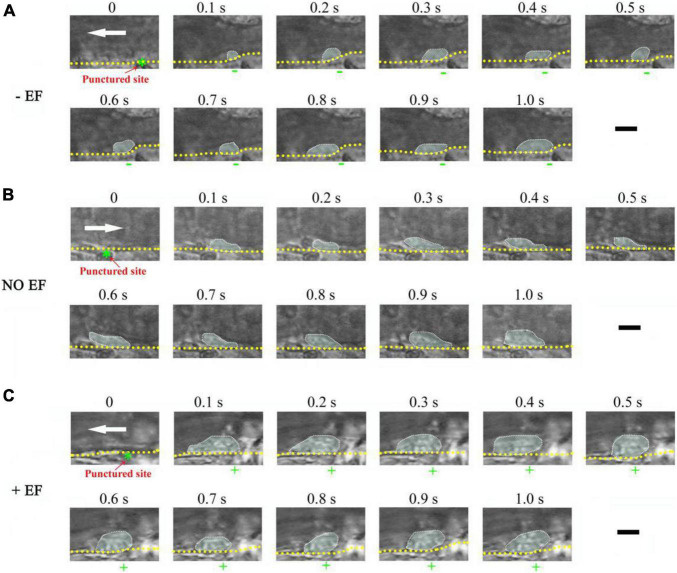
Representative time series of images showing the effect of –EF and +EF on platelet deposition onto the injured surface of the mesenteric blood vessel. **(A)** Punctured injury induced platelet deposition when a cathode with TVEP_cathode_ of –5.4 mV (EF, –13.0 mV/mm) was placed on the punctured site beforehand (vessel diameter, ∼28 μm). **(B)** Platelet deposition induced by punctured injury in the absence of EF (vessel diameter, 30 μm). **(C)** Punctured injury induced platelet deposition when an anode (green star) with TVEP_anode_ of +6.7 mV (EF, 16.1 mV/mm) was placed on the punctured site beforehand (vessel diameter, 35 μm). The deposited platelets are highlighted and the outer perimeter of the area of the deposited platelets is circled with the dotted marquee according to the previous report (Nesbitt et al., 2009). +, anode. –, cathode. White arrow, direction of blood flow. Yellow dotted lines, boundary of blood vessels. Scale bar, 10 μm.

**FIGURE 3:**
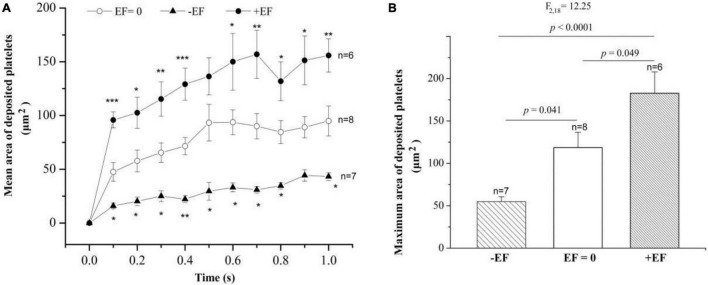
Statistical summary on the effect of –EF and +EF on the platelet deposition onto the injured surface. **(A)** Mean area of deposited platelets at each time point after punctured injury at the condition of default hemostasis (*EF* = 0) and when a cathode (TVEP_cathode_ ∼ –10 mV, EF ∼ – 4.1 mV/mm) or an anode (TVEP_anode_ ∼ +10 mV, EF ∼ +24.1 mV/mm) was placed on the injured site beforehand. Statistical tests are one-way ANOVA, *post hoc* Tukey’s tests. *p < 0.05; **p < 0.01; ***p < 0.001. **(B)** Effect of the electrical signal on the maximal area of deposited platelets within 1 s after the punctured injury (one-way ANOVA, *post hoc* Tukey’s tests).

To study whether the manipulation of electrical signal affects platelet deposition *in vivo*, we initiated platelet deposition by mechanically puncturing the vessel wall of mouse mesenteric vessels with a glass micropipette (tip diameter 4–6 mm). Representative time series of images showing platelet deposition when −EF (cathode on the injured site), +EF (anode on the injured site), and under default hemostasis (no EF) are shown in Figures 2A–C, respectively. These images demonstrated that the deposited platelets were not solely increased but alternatively increased or decreased with time. Comparing the images under default hemostasis, we can find that −EF reduced the platelet deposition, while +EF enhanced the platelet deposition. This is clearly shown in Figure 3A, which is the statistical summary for the mean area of platelet deposition onto the injured site at each time point within 1 s. As shown in the figure, the mean area of the deposited platelet was significantly reduced by –EF, but was increased by +EF relative to that without EF for each time point, respectively. The maximal area of platelet deposition within 1 s (Figure 3B) showed that the mean maximal area within 1 s of punctured injury without EF was 118.6 ± 18.1 μm^2^. When −EF was applied, the maximal size was reduced to 55.0 ± 5.8 μm^2^. In contrast, the maximal size within 1 s of punctured injury was 182.7 ± 25.2 μm^2^ if +EF was applied. These results suggested that the electrical signal could regulate the degree of platelet deposition.

**FIGURE 4:**
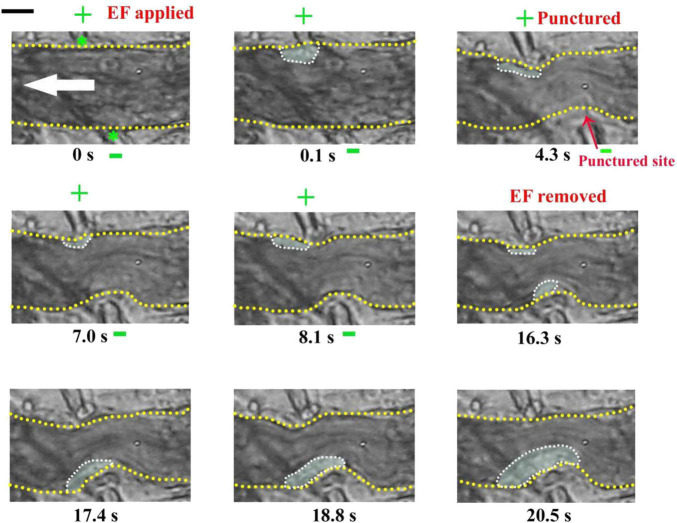
Injury fails to induce platelet accumulation when –EF was applied on the injured site. –EF was applied at *t* = 0 s and removed at *t* = 16.3 s. The vessel with a diameter of ∼23 μm was punctured by a microelectrode at *t* = 4.3 s. +, anode. –, cathode. Green stars, tip of microelectrodes. Dotted marquee, outer perimeter of deposited platelets. Yellow dash lines, boundary of blood vessels. White arrow, direction of blood flow. The results were confirmed by 7 independent experiments. Scale bar, 10 μm.

Interestingly, sometimes, the injury failed to induce platelet deposition if a cathode was placed at the injured site beforehand. For example, platelets did not deposit onto the punctured site from 4.3 to 8.1 s but deposited onto the uninjured area where the anode with TVEP_anode_ of 8.4 mV (EF, 20.2 mV/mm) was placed on the injured site (0.1–8.1 s) (Figure 4). In contrast, when the applied EF was removed at *t* = 16.3 s, the platelet deposition appeared at the punctured site (white dotted marquee from 16.3 to 20.5 s). Meanwhile, the deposited platelets at the opposite side where the anode was once placed floated away. These data indicated that the polarity of the electrical signal plays an important role in platelet deposition onto the injured site.

### The Outer Layer of the Deposited Platelets Could Be Removed if a Cathode Was Placed at the Injured Site After the Injury

**FIGURE 5:**
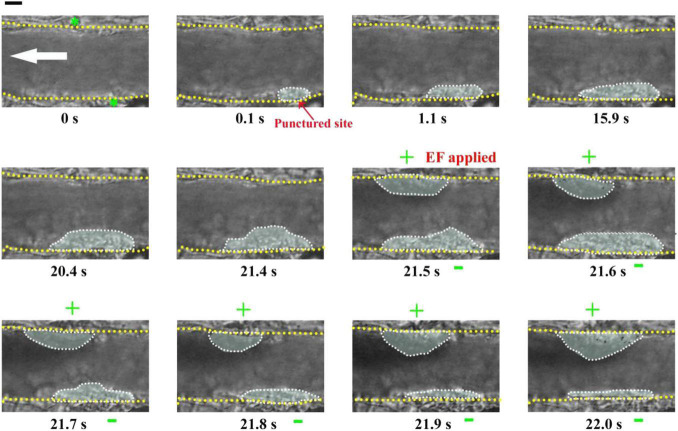
Electrical signal could not eliminate platelets that had already been accumulated in the core region of the injury. First panel (*t* = 0 s): Configuration for applying EF on the mesenteric vessel with a diameter of ∼39 μm. Yellow dotted lines, boundary of the vessel walls. White arrow, direction of blood flow. Green stars, tip of microelectrodes. Dotted marquee, outer perimeter of deposited platelets. The vessel wall was punctured at *t* = 0 s by a microelectrode. EF was applied at *t* = 21.5 s with TVEP_anode_ of +18.6 mV (EF, 44.8 mV/mm). +, anode. –, cathode. Scale bar, 10 μm. The results were reproduced in 9 independent experiments.

To investigate whether the electrical signal could affect the injury-induced platelet deposition, we initiated platelet deposition by puncturing the vessel wall of the mouse mesenteric vessel with a glass microelectrode. At this condition without an applied EF (default hemostasis), the platelet deposition clearly appeared in the injured area after the punctured injury (white dotted marquee from 0.1 to 21.4 s) (Figure 5). Interestingly, the inner layer of the deposited platelets could not be removed even a cathode of the applied EF was placed on the injured site (from 21.5 to 22 s). At the same time, accompanying with the reduction of the size of the deposited platelets at the injured site, we observed that platelets accumulated at the opposite side where the anode was placed, even though there was no vascular injury. These observations suggested that the electrical signal could eliminate platelets on the surface and suppress further platelet deposition, but could not eliminate platelets that had already been deposited in the core region of the injury.

### Electrical Signal Could Drive Platelet Deposition Beneath the Anode Even on Uninjured Blood Vessels

**FIGURE 6:**
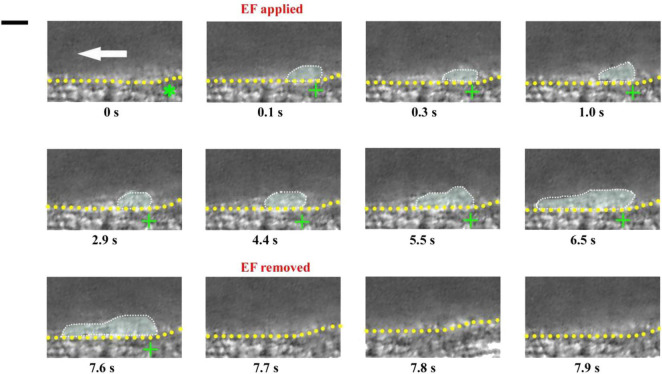
Electrical signal causes platelet accumulation in uninjured mesenteric vessels. Intravital image sequence with TVEP_anode_ of +6.9 mV (EF, 16.6 mV/mm) in an uninjured mesenteric vessel (diameter ∼ 55 μm). The EF was applied at *t* = 0 s and removed at *t* = 7.7 s. Green stars, tip of microelectrodes. Yellow dotted lines, boundary of blood vessels. White dotted marquee, deposited platelets. Scale bar, 10 μm. White arrow, direction of blood flow. +, anode. –, cathode. The results were reproduced in 9 independent experiments.

If an electrical signal *per se* could drive platelet deposition, its action should not be affected by other injury-induced biophysical or biochemical factors. This means that an EF could also drive platelet deposition even on uninjured blood vessels. To test this inference, we carried out experiments on mouse mesenteric vessels that were not injured. There was no platelet deposition before the application of an EF (*t* = 0, Figure 6). In contrast, small platelet deposition was present when the anode with TVEP_anode_ of +6.9 mV (EF, 16.6 mV/mm) was applied in spite of no vascular injury (*t* = 0.1 s, Figure 6). After that, the area of the deposited platelets was gradually increased to some extent. Notably, the deposited platelets were floating away once the applied EF was removed (*t* = 7.7 s Figure 6), indicating that electrical signal *per se* could cause platelet deposition at millisecond time scale even in the uninjured blood vessels *in vivo*.

**FIGURE 7:**
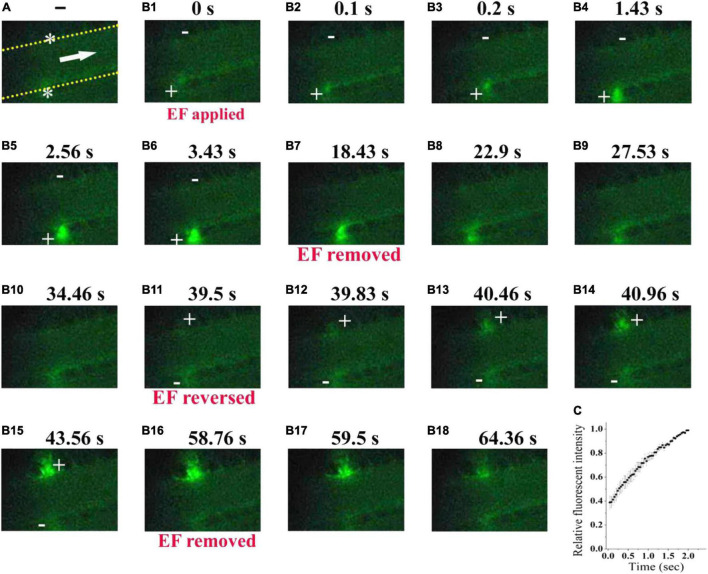
Electrical signal directs fluorescent-labeled platelets to accumulate beneath the anode. **(A)** Configuration for applying EF on an uninjured mesenteric vessel with a diameter of 45 μm. Yellow dotted lines, boundary of vessel walls. White arrow, direction of blood flow. White stars, tip of microelectrodes. **(B_1_–B_18_)**. Representative intravital image sequence of fluorescent-labeled platelet deposition (*n* = 8). The electric field was applied at *t* = 0 s **(B_1_)**, removed at *t* = 18.43 s **(B_7_)**, reversed at *t* = 39.5 s **(B_11_)**, and removed again at *t* = 58.76 s **(B_16_)**. Scale bar, 10 μm. +, anode. –, cathode. **(C)** Summary on the relative fluorescent intensity (% of maximum) during the first 2 s of applying EF (*n* = 6).

To further confirm the ability of electrical signal to drive platelet deposition onto the uninjured vessel wall, we labeled platelets with fluorescent dye (Figure 7A). With TVEP_anode_ of +16.5 mV 750 (EF, 39.7 mV/mm, Figure 7B_**1**_), the fluorescent-labeled platelet accumulation clearly appeared underneath the anode after 100 ms of the EF application (Figure 7B_**2**_), and the fluorescent intensity beneath the anode was gradually increased with time even without a vascular injury (Figures 7B_**2**_–B_**6**_,C). In contrast, the fluorescent intensity was gradually decreased when the EF was removed (Figures 7B_**7**_–B_**10**_). Furthermore, when the polarity of the EF was reversed (Figure 7B_**11**_), the fluorescent intensity was gradually increased on the opposite side of the vessel (Figures 7B_**11**_–B_**15**_) and gradually decreased when the EF was removed (Figures 7B_**16**_–B_**18**_). These observations indicated that the electrical signal *per se* was sufficient to induce platelet deposition.

### Electrical Signal-Triggered Platelet Deposition Is Significantly Reduced by Neuraminidase Treatment

**FIGURE 8:**
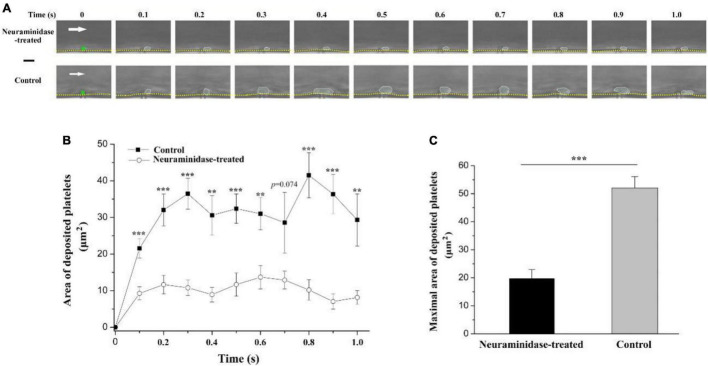
Effect of neuraminidase treatment on EF-induced platelet deposition in microfluidic channel. **(A)** Representative time series of images on deposited platelets under shear rate of 200 s^–1^ in the period of 1 s beginning from the start of the blood flow for neuraminidase-treated (1 U/ml) group (*EF* = 19.7 mV/mm) and control group (*EF* = 20.1 mV/mm). White dotted marquee, perimeter of deposited platelets. Green star, tip of microelectrode. Yellow dash lines, boundary of microfluidic channel. White arrow, direction of blood flow. Bar = 10 μm. **(B)** Statistical summary on the time courses of areas of deposited platelets for the group treated with (*n* = 10) or without (*n* = 9) neuraminidase (1 U/ml) under shear rate of 200 s^–1^. The average values of EF for the group treated with or without neuraminidase were 20.6 ± 1.2 and 21.4 ± 1.3 mV/mm, respectively. **(C)** Statistical summary on the maximal area of deposited platelets for the group treated with or without neuraminidase. Unpaired Student’s *t*-test was used for statistical analysis. **p < 0.01; ***p < 0.001.

The above results suggested that the underpinning mechanism of the electrical signal should be based on the surface charge of platelets. However, the platelet deposition onto the injured surface *in vivo* is a complex phenomenon. It has been reported that treatment with neuraminidase leads to a reduction in the electrokinetic charge (Seaman and Vassar, 1966) or electrophoretic mobility of platelets (Nomura and Takagi, 1974). Therefore, to further verify whether the effect of electrical signal is based on the surface charge of platelets, we reduced platelet surface charge by using neuraminidase to remove sialic acid residues and carried out experiment in a microfluidic system. There were no platelets on the tip of the anode without EF. By applying EF, we observed that platelets deposited onto the tip of the anode in the microfluidic channel within 100 ms (fps = 10) no matter whether the blood samples were treated with neuraminidase or not. However, the average area of the deposited platelets was significantly reduced by neuraminidase treatment at every time points comparing with that without neuraminidase treatment (Figure 8). The maximal area of the deposited platelets within 1 s was 19.9 ± 3.1 μm^2^ for neuraminidase-treated group, while it was 52.1 ± 4.0 μm^2^ for the group without neuraminidase treatment. These results provided further evidence that the effect of EF on platelet deposition was due to the surface charge of platelets.

## Discussion

The possible relationship between electrical signal and thrombus formation has been studied since more than half a century ago (Sawyer et al., 1953, 1960; Sawyer and Pate, 1953; Sawyer and Deutsch, 1955; Sawyer and Deutch, 1956; Schwartz, 1959; Schwarz and Richardson, 1961), but is neglected at the present time. From 1950s to 1970s, Sawyer and colleagues carried out a series of researches to show that electrical signal could not only trigger but also prevent thrombosis. However, most of their studies focused on how strong the electrical signal should be to cause thrombus formation. These experiments revealed that thrombosis occurs on electrodes in most cases when the potential of the electrode is equal to or greater than +300 mV (Sawyer et al., 1965; Sawyer and Sprinivasan, 1967). However, such electrical signal might be too strong. Since most of the blood cells carry negative surface charges (Born and Palinski, 1985), it is likely that a strong electrical signal (equal to or greater than +300 mV) could result in the aggregation of blood cells, including red blood cells, white blood cells, and platelets, to the positive electrode.

There were no platelets on the tip of the microelectrode without EF. If an EF was applied, platelets were deposited onto the tip of the anode within 100 ms (fps = 10) in most of the experiments. It is noteworthy that the area of the deposited platelets at 100 ms was about half to two-third of the maximal area (Figures 2C, 3A, 6). Electrostatic interaction is mediated by an electric field, which takes action, not instantaneous, but propagates in time in a similar manner to that of light. Therefore, the applied electric field can take action immediately on the platelets from the moment of its application. That is, the platelets attracted by the tip of the anode microelectrode should be increased with time from the onset of the applied EF. As demonstrated in Figure 3A, the area of the deposited platelets reached about two-third of the maximal area after the first 100 ms. However, in the flowing blood, the larger the area of the deposited platelets, the larger the shear force on them. So, we observed that the area of the deposited platelets was not solely increased but was alternatively increased or decreased with time (Figures 2, 6, 8).

Circulating platelets are involved in different processes such as triggering inflammation, fighting microbial infection, promoting tumor metastasis, and embryonic blood/lymphatic vessel separation. Nevertheless, their principal function still remains stopping hemorrhage following vascular injury by forming platelet plug in primary hemostasis. Therefore, it is desired that only platelets but no other blood cells could be regulated to deposit onto the injured site during the process of platelet plug formation. The sizes of RBC and leukocyte are much larger than that of platelets, the mean diameter of which is 1.50 μm for mice (Tsakiris et al., 1999; Thon and Italiano, 2010). Therefore, based on the difference in sizes between platelets and other blood cells, we found that the deposited cells at the injured site were predominantly platelets when a smaller electrical signal (e.g., TVEP_anode_, less than 20 mV) was applied. This might be due to the two following reasons: (1) The charge density of platelets is larger than that of other blood cells (Greger and Thomas, 1960; Born and Palinski, 1985); (2) The platelet margination effect, i.e., the number of platelets in the vicinity of the vessel wall is much more than that in the center of the vessel (Tangelder et al., 1985; Tokarev et al., 2011a,b). It means that electrical signal could exert much larger force on the platelets than on other blood cells near the blood vessel wall. Therefore, platelets rather than other blood cells are overwhelmingly deposited onto the injured site when a smaller electrical signal was applied. For example, we found that the smaller electrical signal with TVEP_anode_ of less than 20 mV could only cause transient platelet deposition, as the deposited platelets were washed away if the applied EF was removed. Thus, our data suggest that manipulation of the electrical signal might be a useful method to regulate platelet deposition onto the vascular vessel wall.

## Data Availability Statement

The raw data supporting the conclusions of this article will be made available by the authors, without undue reservation.

## Ethics Statement

The animal study was reviewed and approved by Animal Care and Use Committee of Xiamen University.

## Author Contributions

ZQ designed the research. MW performed the experiments. All authors analyzed the data and wrote the manuscript.

## Conflict of Interest

The authors declare that the research was conducted in the absence of any commercial or financial relationships that could be construed as a potential conflict of interest.

## Publisher’s Note

All claims expressed in this article are solely those of the authors and do not necessarily represent those of their affiliated organizations, or those of the publisher, the editors and the reviewers. Any product that may be evaluated in this article, or claim that may be made by its manufacturer, is not guaranteed or endorsed by the publisher.
